# Unraveling the roles of pressure, oxidation state, and morphology in CO_2_ electroreduction to C_2+_ gaseous products over copper oxides†

**DOI:** 10.1039/d4na01019a

**Published:** 2025-02-25

**Authors:** Asghar Ali, Ali S. Alnaser

**Affiliations:** a Materials Science and Engineering Program, Materials Research Center, College of Arts and Sciences, American University of Sharjah Sharjah UAE aalnaser@aus.edu; b Department of Physics, College of Arts and Sciences, American University of Sharjah Sharjah UAE; c Materials Research Center, American University of Sharjah Sharjah UAE

## Abstract

This study provides compelling experimental evidence of the synergistic effects of reaction pressure, oxidation state, and catalyst morphology on the C_2+_ selectivity of copper (Cu) oxide catalysts in electrochemical CO_2_ reduction (ECR). We employed femtosecond laser structuring and thermal treatments to synthesize Cu(0), Cu(i), Cu(ii), and a mixed oxidation state catalyst Cu(*x*) with characteristic micro- and nano-morphologies. The optimal CO_2_ pressure for maximizing C_2+_ productivity in aqueous bicarbonate media was established by assessing the reaction products at different imposed pressures in a custom-designed, pressurizable two-compartment cell. Among Cu(0), Cu(i), and Cu(ii), thermally produced Cu(i) was the only unstructured catalyst exhibiting ethylene gas-phase selectivity. Nanostructuring enhanced the C_2+_ selectivity such that all three oxidation states could produce ethylene. More importantly, the nanostructured Cu(*x*) comprising well-dispersed Cu(0), Cu(i), and Cu(ii), exhibited ethylene as well as ethane production – a characteristic associated with the synergistic effects of undercoordinated Cu states in stabilizing reaction intermediates and facilitating charge transfer to yield longer C_2+_ products. This work provides important insights into the key factors influencing C_2+_ selectivity in Cu-based catalysts, establishing the basis for an informed design to yield high-energy density products.

## Abstract 2

This experimental investigation presents compelling evidence of the synergistic effects of reaction pressure, oxidation state, and catalyst morphology on the C_2+_ selectivity of copper oxide catalysts in electrochemical CO_2_ reduction.

## Graphical abstract

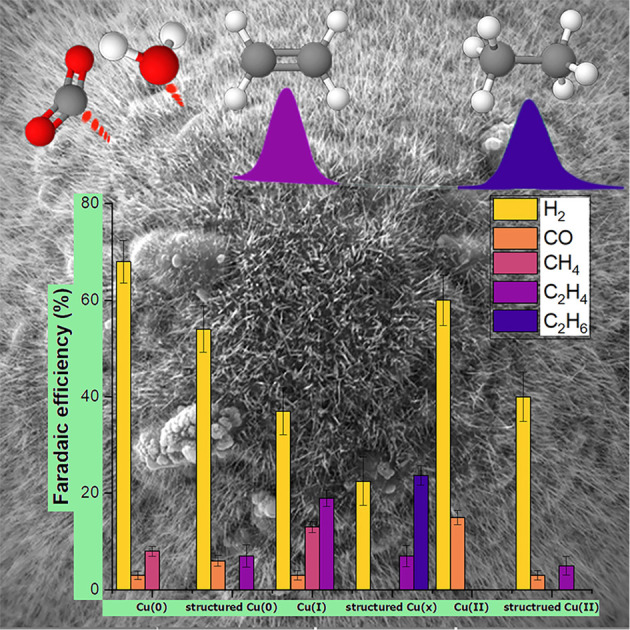

## Introduction

Electrochemical CO_2_ reduction (ECR) is a promising approach to mitigate anthropogenic CO_2_ and simultaneously produce fuels and other value-added chemicals by electrocatalytically reducing CO_2_. ECR is a sustainable net-zero emissions strategy for climate remediation, energy security, and long-term energy storage. Among the array of chemicals producible through ECR, some important products are carbon monoxide (CO), formate (HCOO^−^), methane (CH_4_), methanol (CH_3_OH), ethylene (C_2_H_4_), ethane (C_2_H_6_), ethanol (C_2_H_5_OH), and propanol (C_3_H_7_OH). The multi-carbon (C_2+_) products, which comprise two or more carbon (C) atoms such as ethylene, ethanol, propanol, *etc.* are attractive due to their high-energy densities, easy utilization, and industrial relevance.^1–8,9^

The surface coverage by the ECR intermediates is crucial in dictating the selectivity of a catalyst. The reaction rate and selectivity of ECR are intricately linked to the surface coverage by CO_2_.^10^ The local CO_2_ concentration along the electrode surface affects the coverage by reaction intermediates such as *CO_2_, *CO, and *H. Thus, the activity and selectivity of ECR are strongly influenced by CO_2_ availability.^11^ Among the strategies adopted to improve CO_2_ availability at the electrode is the use of ionic liquids instead of water-based electrolytes.^12^ Due to their high CO_2_ absorption capacity, thermodynamic stability, and non-volatility, ionic liquids are employed for CO_2_ capture.^13,14^ Ionic liquids have garnered significant attention in ECR owing to their high ionic conductivity, ability to stabilize reaction intermediates, electrochemical stability, capacity to lower overpotentials, and structural tunability. These physicochemical attributes position ionic liquids as critical for integrated CO_2_ capture and conversion.^12^ However, their high cost mainly prohibits their large-scale industrial adoption. In contrast, elevating the pressure is a relatively more affordable strategy to improve CO_2_ solubility in aqueous electrolytes.^15^ Pressure incursions in aqueous bicarbonates can also suppress the competing hydrogen evolution reaction (HER) and thus improve ECR selectivity.^11^ However, a moderate local CO_2_ concentration is crucial for realizing high C_2+_ selectivity.^16^ It is therefore imperative to estimate the optimum CO_2_ pressure required for realize high C_2+_ selectivity on any desired catalyst in bicarbonate media.

Copper (Cu)-based catalysts are known for selectively yielding high-energy density C_2+_ products.^1,15^ The C_2+_ selectivity of Cu-based catalysts is attributed to their intermediate binding interaction with key reaction intermediates, which in turn depends on the electronic structure and oxidation state of Cu.^17,18^ Cu catalysts typically exhibit Cu(0), Cu(i), and Cu(ii) oxidation states.^19^ Cu(0) and Cu(i) are reportedly more attractive for ECR due to their moderate binding energies towards reaction intermediates such as adsorbed CO (*CO), facilitating C–C coupling.^17,18^ Recent studies also suggest that the coexistence of Cu(0) and Cu(i) centers in close vicinity is important for realizing C–C coupling.^17^ Yet there are studies that suggest that Cu(i) is the actual catalytic center responsible for C–C coupling and stabilizing the intermediates,^20–23^ and Cu(0) and Cu(ii) might transform into Cu(i) for participating in certain catalytic reactions.^24^ Nonetheless, these studies are often performed on catalysts with varying nanostructures, and the role of catalyst morphology is frequently overlooked, resulting in disparities when interpreting the observed results.

The structure and morphology of the catalysts are also key factors in determining ECR selectivity.^15^ Nanomorphology promote ECR through improved selectivity.^25^ This is because the coordination geometry affects the electronic structure of the catalyst, thus influencing its selectivity.^23^ Smaller sized nanofeatures result in undercoordination and expose high-index facets, which promote C–C coupling by stabilizing the reaction intermediates.^26,27^ In this regard, steps, nanofeatures, hierarchical structures, nanocubes, and nanograin boundaries are known to promote C_2+_ selectivity.^28,29^ Uneven nanosheet structures can also lead to electron perturbation and modified electron distribution, which can affect the adsorption kinetics and module selectivity. Cubic, hexarhombic, dodecahedral, and octahedral Cu nanoparticles have been shown to significantly affect the Faradaic efficiency (FE) of both gas and liquid products.^30^ Due to the undeniable importance of catalyst morphology in determining selectivity, the morphological effects should be considered in correlation with the oxidation state effects on ECR selectivity. Moreover, catalyst stability is also critical as the oxidation state and morphology may change under ECR conditions, thus influencing the product distribution.^17,18^

This study experimentally explores the complex relationship between reaction pressure, oxidation state, and catalyst morphology in influencing the C_2+_ selectivity of Cu-based electrocatalysts. Through the application of femtosecond laser surface structuring and thermal treatment procedures, we synthesize Cu(0), Cu(i), Cu(ii), and mixed oxidation state Cu(*x*) catalysts of characteristic micro and nano-morphologies. The optimum pressure conditions for realizing high C_2+_ selectivity are investigated in a custom-designed two compartment reactor in bicarbonate media. A comprehensive analysis of the effects of Cu oxidation states in correlation with catalyst morphologies on the C_2+_ selectivity is provided. The role of undercoordinated mixed oxidation states of Cu(0), Cu(i), and Cu(ii) in ethane selectivity is investigated. Lastly, the stability of the catalysts under operating conditions is evaluated. This study provides important experimental evidence of the key factors influencing the C_2+_ selectivity of Cu-based catalysts, providing valuable insights for the informed design of advanced Cu-based electrocatalysts.

## Experimental

Copper substrates (1 mm thickness) were cut into 1.7 cm diameter discs. The Cu discs were subsequently ground and polished with a grinder and polisher (Forcipol 102, Metkon). These discs were subsequently cleaned with deionized water, ethanol, and acetone. For surface structuring, these samples were subjected to femtosecond laser scanning. A ytterbium-based femtosecond laser (AFSUFFL-300-2000-1030-300, Active Fiber Systems GmbH) with a central wavelength of 1030 nm, 40 fs pulse duration, and a 50 kHz repetition rate was used. A Gaussian beam was directed through a half-waveplate (*λ*/2) plate and a thin-film polarizer to adjust and achieve an output power of 5 W. The beam was focused onto a 50 μm spot size using an *f* − *θ* lens on a computer programmable scan head. The beam was scanned at a speed of 10 mm s^−1^, in either a single parallel or a 5 crosshatch scan configuration. The line spacing was 20 and 70 μm for the parallel and crosshatch scans, respectively. For introducing oxides, structuring was performed in air, otherwise it was conducted in an Ar filled chamber to avoid oxidation. To further reduce samples, electrochemical reduction was conducted at −5 mA cm^−2^ for 3 minutes. Thermal treatments were carried out in a tube furnace (OTF-1200X, MTI) at 220 °C and 350 °C.

The morphology of the samples was characterized using a scanning electron microscope (VEGA-3 LMU, TESCAN). Elemental composition was determined using an energy dispersive X-ray spectroscope (EDX, INCAx-act, Oxford Instruments) fitted onto the scanning electron microscope. Further chemical insights were acquired with a confocal Raman imaging microscope (alpha300 R, WITec, Germany). A 10× objective lens was used to focus a 532 nm green laser onto the sample. Further information about the oxidation states was acquired with X-ray photoelectron spectroscopy (Nexsa G2, Thermo Scientific) using Al Kα X-rays (*hv* = 1486.6 eV). The resolution of the high resolution spectra was 0.1 eV and the spectra provided here are the average counts per second taken over 5 scans. XPS depth profiles were recorded after etching with Ar^+^ ions using a monoatomic etcher (MAGCIS). Baseline correction, curve fitting, and area integration were performed with OriginPro 2021b (version: 9.8.5.204 (Academic)).

Electrochemical measurements were performed in a custom-designed Teflon lined two compartment stainless steel reactor with a Nafion-212 membrane separating the two compartments. Ag/AgCl (3 M) and platinum wire were used as the reference and counter electrodes, respectively. For saturation and pressure balancing, the catholyte and anolyte compartments, each containing ∼100 ml of 0.1 M NaHCO_3_, were sparged independently with pressurized CO_2_ at a flow rate of ∼30 sccm. The catholyte was sparged using a specially designed 3D-printed sparge head submerged in the solution. The pressure was varied in the range of 1–4.5 bar (absolute). For galvanostatic measurements, the current was maintained at −10 mA cm^−2^, whereas potentiostatic measurements were carried out at −2 V *vs.* Ag/AgCl (3 M). An Autolab VIONIC potentiostat/galvanostat (Metrohm) was employed. Unless otherwise specified, all reported potentials are relative to Ag/AgCl (3 M), without conversion to the reversible hydrogen electrode (RHE). The gas product line from the cathode compartment was connected to an Agilent 990 micro GC for product analysis. The GC uses thermal conductivity detectors (TCD) to quantify H_2_, CO, CH_4_, C_2_H_4_, and C_2_H_6_. The reproducibility of the GC measurements was constantly monitored with a standard gaseous mixture. Each result reported here has been reproduced at least three times. FE was calculated using the following equation:FE (%) = *nZF*/*Q*where *n* is the number of moles of the product under consideration, *Z* is the number of electrons transferred to produce a product molecule, *F* is the Faraday constant (96 485 C mol^−1^), and *Q* is the total charge consumed during the reaction.

## Results and discussion

**Fig. 1:**
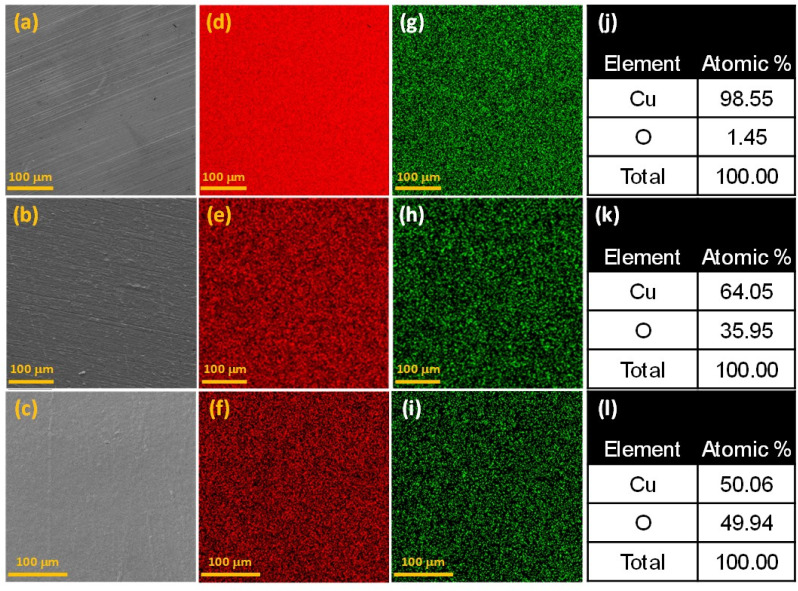
(a–c) SEM micrographs of unstructured Cu surfaces subjected to different thermal treatments: (a) untreated, (b) treated at 220 °C, and (c) treated at 350 °C. EDS maps illustrating the distribution of (d–f) Cu and (g–i) O on the corresponding surfaces shown in (a–c). (j–l) Tables summarizing the percentage elemental composition of Cu and O along the corresponding (a–c) surfaces.

It is known that metallic Cu, when thermally treated in air at lower temperatures around 220 °C yields only Cu(i) oxide, whereas Cu(ii) starts to form only above 320 °C.^31^ Based on these findings, we produce Cu(i)- and Cu(ii)-rich catalysts *via* simple furnace treatments. Fig. 1 shows flat Cu substrates, thermally treated at different temperatures to realize the desired unstructured Cu(0), Cu(i), and Cu(ii) oxidation states. Fig. 1(a) present the SEM image of a ground and polished Cu surface with the corresponding EDS maps shown in Fig. 1(d and g). The elemental composition (Fig. S1†) summarized in Fig. 1(j) reveals that mechanical grinding and subsequent polishing removed most of the surface oxides from the Cu surface to form Cu(0). Since the sample is mostly metallic Cu, based on the oxidation state, we designate this sample as Cu(0). The surface after thermal treatment at 220 °C is shown in Fig. 1(b) and the corresponding elemental maps are shown in Fig. 1(e and h). The elemental composition (Fig. S2†) summarized in Fig. 1(k), depicts oxidation of the surface with thermal treatment. We designate this unstructured sample as Cu(i). The surface of polished Cu after thermal at 350 °C is shown in Fig. 1(c) with the corresponding elemental maps shown in Fig. 1(f and i). The elemental composition (Fig. S3†) outlined in Fig. 1(l), indicates strong oxidation of the Cu surface. Based on the treatment temperature and the resulting Cu : O ratio, the sample will henceforth be referred to as Cu(ii).

It is noteworthy that the thermal treatments affect not only the oxidation but also the surface nanomorphology. Treatment at 220 °C roughened the surface with some nanofeatures appearing on the surface due to surface reconstruction upon oxidation (Fig. S4(a)†). Similarly, treatment at 350 °C resulted in CuO nanowires along the surface (Fig. S4(b)†). These CuO nanowires are discussed in more detail in the following discussion on catalyst morphology.

**Fig. 2:**
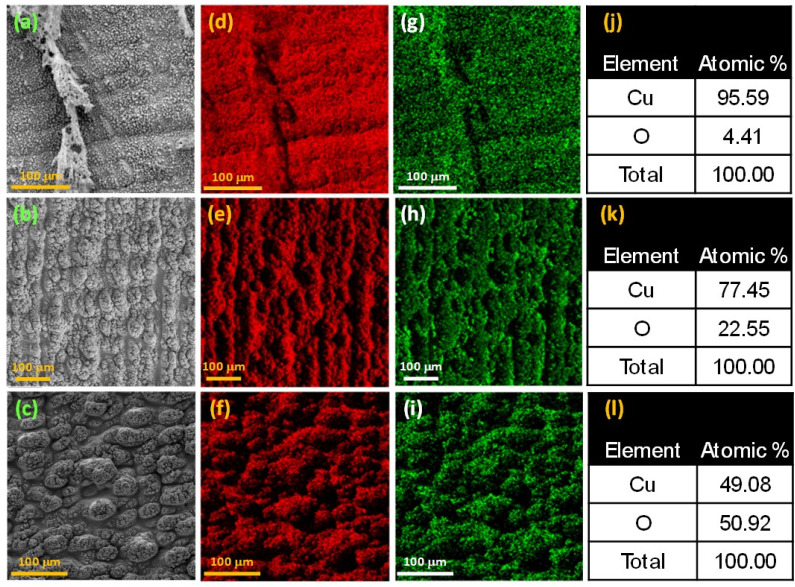
(a–c) SEM micrographs of femtosecond laser structured Cu (with 20 μm parallel line spacing) followed by thermal treatment: (a) structured in Ar and no thermal treatment, (b) structured in air and no thermal treatment, (c) structured and thermally treated at 350 °C in air. EDS maps illustrating the distribution of (d–f) Cu and (g–i) O on the corresponding surfaces shown in (a–c). (j–l) Tables summarizing the percentage elemental composition of Cu and O along the corresponding surfaces shown in (a–c).

Different surface morphologies were produced with femtosecond laser surface structuring and subsequent thermal treatments. Fig. 2(a) depicts a highly roughened Cu surface produced by structuring in Ar. Its elemental distribution maps are shown in Fig. 2(d and g), with the elemental composition (Fig. S5†) summarized in Fig. 2(j). The ∼4.4 at% O content indicates that the microstructure is primarily composed of metallic Cu(0), and will be referred to as s-Cu(0) (here, s denotes laser-structured). Similarly, Fig. 2(b), depicts a cauliflower-shaped microstructure produced with femtosecond laser structuring in air. The corresponding elemental distribution maps are shown in Fig. 2(e and h), whereas the elemental composition (Fig. S6†) is summarized in Fig. 2(k). Due to the unknown oxidation state of this sample, we refer to it as s-Cu(*x*) (*x* denotes the unknown oxidation state). To create a highly rough Cu(ii) surface, the laser structured Cu (mentioned above) was thermally treated at 350 °C in air. The corresponding morphology and elemental maps are shown in Fig. 2(c), (f) and (i), respectively. Its elemental composition (Fig. S7†) is summarized in Fig. 2(l). The thermal treatment temperature and the resulting Cu : O indicate Cu(ii) oxidation. This sample will be called s-Cu(ii) through the remainder of this study.

**Fig. 3:**
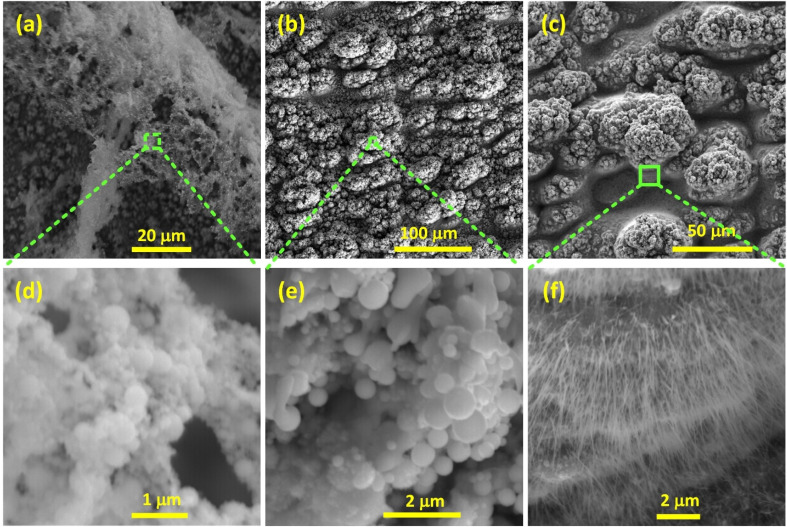
(a–c) SEM micrographs of femtosecond laser-structured Cu (20 μm parallel line spacing), with (d–f) corresponding zoomed-in views. (a–d) Cu structured in Ar, showing spherical submicron and nanoparticles. (b–e) Cu structured in air, showing spherical submicron and nanoparticles. (c–f) Cu structured in air and annealed at 350 °C, depicting nanowire-decorated microfeatures.

To explore the nanomorphology of the surface, further SEM analysis was performed (Fig. 3). Fig. 3(a, d) show s-Cu(0) with hierarchical surface structures, comprising Cu(0) submicron particles and nanospheres superimposed on the microstructures. Similarly, Fig. 3(b, e) show s-Cu(*x*), depicting cauliflower-shaped microstructures decorated with spheres of submicron and nano-sizes. Similarly, Fig. 3(c, f) shows s-Cu(ii) (350 °C treated s-Cu(*x*)), depicting the cauliflower shaped microstructures adorned with thermally grown Cu(ii) nanowires.

**Fig. 4:**
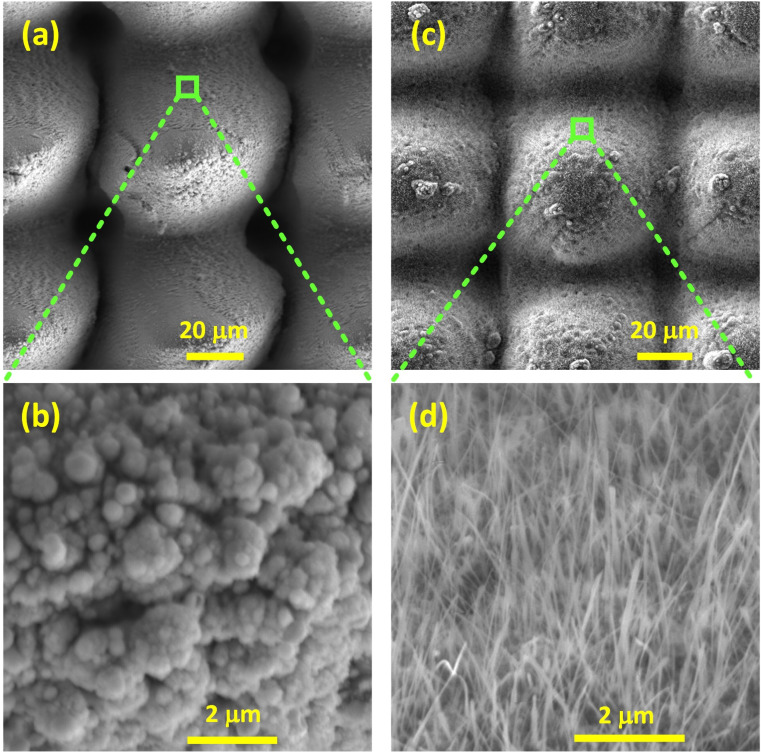
SEM micrographs of femtosecond laser structured Cu (70 μm crosshatch line spacing) with (a and b) no thermal treatment depicting submicron and nanospheres decorating microdomes, and (c and d) thermally treated at 350 °C in air, showing nanowires decorating the microdomes.

Dome microstructures decorated with nanofeatures were produced with femtosecond laser scanning in a crosshatch configuration (Fig. 4). Fig. 4(a and b) show Cu structured in air without any subsequent thermal treatment. Hierarchical structures comprising submicron and nano-sized spheres of substoichiometric O (Fig. S8†), decorating the dome microstructures could be witnessed. Similarly, Fig. 4(c and d) represent Cu crosshatched as described above, followed by annealing at 350 °C in air. Hierarchical structures comprising Cu(ii) (Fig. S9†) nanowires decorating the microdomes were observed. Due to the peculiar dome structured (ds) morphology and the O content, these samples will be referred to as ds-Cu(*x*) (where *x* represents substoichiometry) and ds-Cu(ii) throughout this study.

The nanowires observed above, are actually CuO crystals, which only appear at higher temperatures (350–964 °C). CuO nanowire growth is not sustainable below 350 °C, and typically Cu_2_O forms at ∼250 °C.^32,33^ Upon heating, Cu first oxidizes into Cu_2_O, and if the temperature is feasible (≥350 °C), only then Cu_2_O further oxidizes into CuO.^32–36^ This is why we only observed Cu_2_O nanospheres while treating Cu(0) at 220 °C. The nanowire growth is governed by the diffusion of Cu from the underlying substrate and O from the atmosphere. Upon heating, Cu atoms diffuse to the CuO/Cu_2_O interface and are exchanged between the equilibrating CuO and Cu_2_O phases. The Cu atoms continue hopping between the two phases until they encounter O diffusing from the atmosphere, eventually forming stable CuO; this way the nanowire keeps on growing. CuO remains stable in the O rich region above the interface, whereas Cu_2_O is more stable in the relatively lower O content region below the interface.^33^

**Fig. 5:**
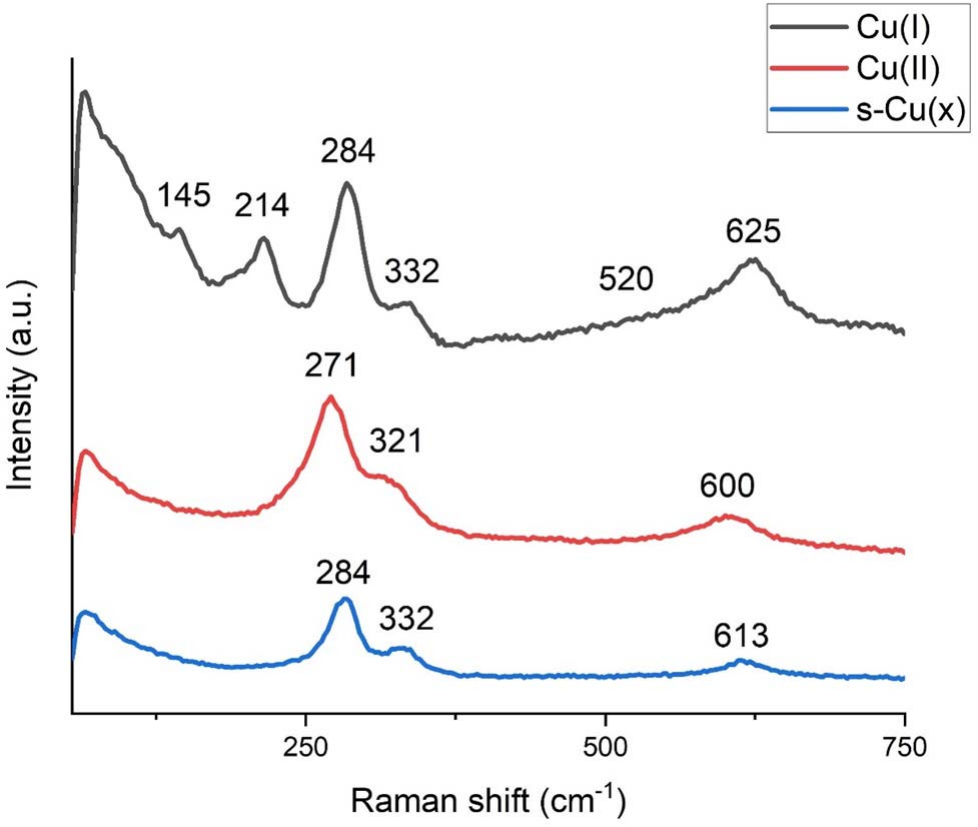
Raman shift analysis of samples predominantly composed of Cu_2_O, as Cu(i), CuO, as Cu(ii), and laser structured Cu with mixed oxidation states, s-Cu(*x*).

The surface chemistry was further elucidated through Raman shift analysis. Fig. 5 compares the Raman shift spectra of Cu-derived samples processed in air, *i.e.*, Cu(i), obtained by treating Cu at 220 °C; Cu(ii), obtained by treating Cu at 350 °C; and s-Cu(*x*), the laser-structured Cu. The broad hump around 520 cm^−1^ is the only band with T_2g_ symmetry and is associated with the presence of Cu_2_O in the Cu(i) sample. The broad hump indicates structural disorder in Cu_2_O, leading to the emergence of additional peaks that are typically absent in well-ordered Cu_2_O. In fact, Cu_2_O is known to reveal Raman forbidden bands and that is why several other bands associated with Cu_2_O were identified.^37^ Among the other peaks associated with Cu_2_O are those at 145 and 214 cm^−1^.^38^ The peak around 284 cm^−1^ appears as a convoluted peak with several subpeaks and may be attributed either to the second order overtone of Cu_2_O or to CuO; however, the peak at around 332 cm^−1^ suggests that the 288 cm^−1^ peak is probably due to CuO.^39^ The broad convoluted band around 625 cm^−1^ is even more interesting. There are instances in the literature suggesting that this band is due to Cu_2_O,^40,41^ whereas others attribute it to CuO.^39,42,43^ Due to the considerable number of peaks detected for Cu_2_O, we can confidently say that the Cu(i) sample is mainly composed of Cu_2_O, but there is a fraction of CuO also present in this sample.

The Cu(ii) sample (Fig. 5) is relatively straightforward and depicts all the peaks representative of CuO around 271, 321, and 600 cm^−1^. The 271 and 321 cm^−1^ bands exhibit an almost constant shift (∼12 cm^−1^) relative to their corresponding peaks detected in Cu(i); however, the 600 cm^−1^ band is shifted by 25 cm^−1^ from the 625 cm^−1^ peak in Cu(i), implying that this band is more sensitive towards changes in the local chemical environment.

The s-Cu(*x*) (Fig. 5) sample shows the two representative bands of CuO at exactly the same position as those for Cu(i), *i.e.*, at 284 and 332 cm^−1^. However, the band at 625 cm^−1^ in Cu(i) shifted to 613 cm^−1^ in s-Cu(*x*). As discussed above, this band is more sensitive to changes in the local chemical environment, showing that this sample is chemically different from both Cu(i) and Cu(ii). In fact, this Raman band has been observed to shift in the 601–624 cm^−1^ range with adsorbed O.^40,44-46^ As shown later in this work, s-Cu(*x*) is an important ECR catalyst and needs more thorough investigation. Further insights into its oxidation state and adsorbed O were acquired using XPS analysis.

**Fig. 6:**
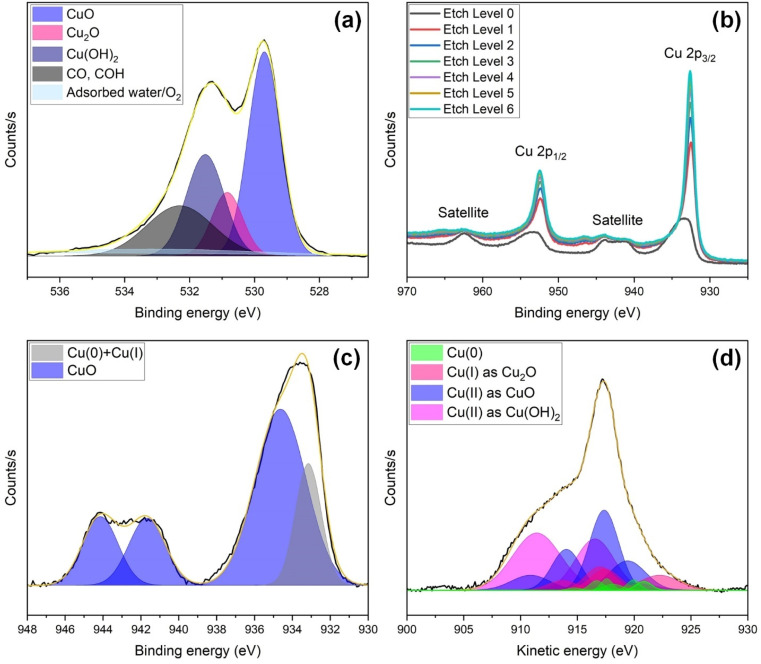
High resolution XPS spectra of s-Cu(*x*) prior to ECR studies, showing (a) O 1s, (b) Cu 2p along with a depth profile, (c) Cu 2p_3/2_, and (d) Cu LMM Auger spectral analysis.

Fig. 6 presents high-resolution XPS analysis of O 1s (Fig. 6(a)), Cu 2p and its depth profile (Fig. 6(b)), Cu 2p_3/2_ (Fig. 6(c)), and Cu LMM Auger (Fig. 6(d)) spectra of the unused laser structured s-Cu(*x*). The O 1s spectrum (Fig. 6(a)) was resolved into 5 peaks at 529.7, 530.8, 531.5, 532.3, and 533.2 eV corresponding to CuO,^47–49^ Cu_2_O,^50^ Cu(OH)_2_,^49,51,52^ C–O/COH,^47,52^ and adsorbed water/O_2_.,^52,53^ respectively. Cu(ii), existing as CuO and Cu(OH)_2_, is apparently more abundant than Cu(i) existing as Cu_2_O. Besides Cu(i), and Cu(ii), the top surface also contains C impurities (organic contaminants) as well as adsorbed water and O_2_. Previously, we have shown that the Raman band shift in the 600–625 cm^−1^ range may be due to this adsorbed O. To further investigate the Cu(0), Cu(i), and Cu(ii) states, we explore the Cu 2p spectrum and its depth profile as the surface is etched under vacuum (Fig. 6(b)). The Cu 2p_1/2_ main peak and its satellite features, along with the Cu 2p_3/2_ main peak and corresponding satellites, are observed within the binding energy range of 950–967 eV and 929–947 eV, respectively.^54,55^ Upon etching, a shift in peak position towards lower binding energies was witnessed, accompanied by a reduction in the satellites and sharpening of the main Cu 2p_1/2_ and Cu 2p_3/2_ peaks. A continuous decrease in Cu(ii) content, likely accompanied by a reduction in O content, is suggested as we move deeper beneath the top surface (Fig. 6(b)).

Although, the Cu 2p_3/2_ peak hardly distinguishes Cu(0) from Cu(i), it can be used to easily quantify Cu(ii) relative to Cu(0) and Cu(i) together. The strong shake-up peaks appearing at 944.1 and 941.7 eV in Fig. 6(c) indicate a considerable Cu(ii) content in the unused s-Cu(*x*). Here, an indicator of the fitting accuracy is the ratio of the areas under the curve of Cu 2p_3/2_ and its satellites, which should be 1.89 for Cu(ii). Our fitting yields the same ratio, thus confirming the reliability of the fitting for quantifying Cu(ii) relative to Cu(0) and Cu(i) combined. Cu(ii) constitutes ∼81.5%, whereas Cu(0) and Cu(i) together constitute ∼18.5% of the total composition. To distinguish the Cu(0) and Cu(i) components, we analyze the Cu LMM spectrum given in Fig. 6(d). The Cu LMM analysis to quantify Cu(0) and Cu(i) is especially useful when the Cu(ii) content is low. This is because the relatively broad Cu(ii) peaks can overshadow the Cu(0) and Cu(i) peaks, as shown in Fig. 6(d). Furthermore, Cu(0) comprises almost seven Cu LMM peaks, whereas, Cu_2_O is represented by four such peaks. Also, when the relative content of Cu(0) and/or Cu(i) is considerably lower than that of Cu(ii), it becomes more challenging to precisely quantify Cu(0) and Cu(i) due to peak overlap with Cu(ii).^56^ The over 80% Cu(ii) determined above is quite large, and although we tried, the exact quantification of Cu(i) relative to Cu(0) remains challenging based on the Cu LMM spectrum. After peak deconvolution, the characteristic peaks of Cu(0) were located at 920.84, 919.94, 918.14, 917.59, 916.7, 914.76, and 911.44 eV. Those of Cu(i) were identified at 922.24, 917.45, 916.98, and 913.69. Cu(ii) in the form of CuO was identified from peaks at 919.42, 917.36, 914.04, and 910.87 eV, whereas Cu(ii) as Cu(OH)_2_ was signified by peaks at 919.61, 916.54, and 911.43 eV. The relative content of Cu(0), Cu(i), and Cu(ii) confirmed from Cu LMM analysis was 5.3%, 16.4%, and 78.3%, respectively. Since the Cu(ii) content calculated from the Cu LMM spectrum (*i.e.*, 78.3%) closely aligns with that from Cu 2p_3/2_ (*i.e.*, 81.5%), the estimated composition of Cu(0) and Cu(i) in the sample is approximately 4% and 16%, respectively. The above discussion leads us to the conclusion that the pre-ECR s-Cu(*x*) comprises mixed oxidation states, with 80% Cu(ii), 16% Cu(i), and 4% Cu(0).

**Fig. 7:**
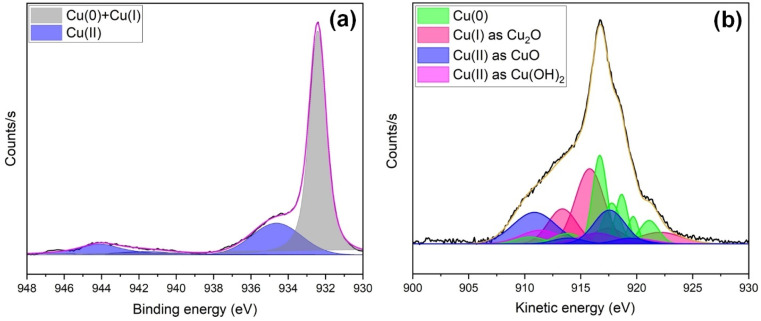
High-resolution XPS spectra of s-Cu(*x*) obtained after electrochemical CO_2_ reduction, presenting (a) Cu 2p_3/2_ and (b) Cu LMM Auger spectral analysis.

To quantify surface changes in oxidation state due to electrochemical CO_2_ reduction, post-ECR XPS analysis of s-Cu(*x*) was also performed. Fig. 7 presents the Cu 2p_3/2_ (Fig. 7(a)) and Cu LMM (Fig. 7(b)) spectra of the s-Cu(*x*) sample acquired after 6 hours of chronoamperometry in NaHCO_3_ at −2 V *vs.* Ag/AgCl (3 M), corresponding to *ca.* −10 mA cm^−2^. Compared to the pre-ECR composition, the Cu 2p_3/2_ spectrum of the post-ECR sample reveals a significant reduction in the Cu(ii) content (934.6 eV) relative to the Cu(0) + Cu(i) content (932.4 eV). The Cu(ii) content drops to ∼34%, whereas Cu(0) + Cu(i) increase to ∼66%. Further insights into the relative composition were provided by the Cu LMM analysis (Fig. 7(b)). The peaks signifying Cu(0) were identified at 921.11, 919.68, 918.65, 917.77, 916.7, 913.8, and 910.48 eV. The characteristic peaks of Cu(i) were located at 922.1, 917.45, 916.45, and 913.36 eV. Similarly, Cu(ii) in the form of CuO was observed at 919.4, 917.57, 913.91, and 910.87 eV. Likewise, Cu(ii) as Cu(OH)_2_ was identified by peaks at 919.8, 916.46, and 911.4 eV. Based on Cu LMM analysis, the post-ECR composition is *ca.* 32% Cu(0), 35% Cu(i), and 33% Cu(ii). The findings show that during ECR, s-Cu(*x*) undergoes reduction from Cu(ii) to Cu(i) and Cu(0) states. Such changes in the oxidation state during ECR, which can influence selectivity, have been witnessed in previous studies as well.^57–59^

**Fig. 8:**
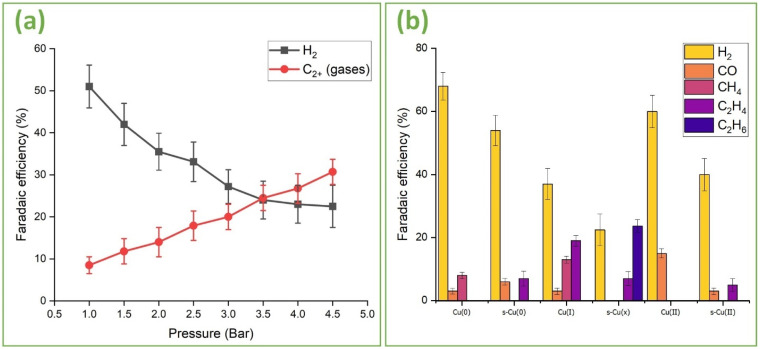
(a) Faradaic efficiency of s-Cu(*x*) for H_2_ and C_2+_ gases as a function of vessel pressure. (b) Faradaic efficiency of all gaseous products observed for unstructured and hierarchically structured Cu(0), Cu(*x*), and Cu(ii) electrodes, respectively.

Fig. 8(a) depicts the effects of pressure incursions on the FE for gaseous products for hierarchically structured s-Cu(*x*) (Fig. 3(b, e)). Increasing the pressure to 4.5 bars triggers suppression of the HER (FE 22%) and promotion of C_2+_ gaseous products (FE 31%). Compared to the FEs at 1 bar, the combined FE of C_2_H_4_ and C_2_H_6_ witnessed an almost 200% increase at 4.5 bar. Meanwhile H_2_ experienced an ∼56% decrease in FE. Almost 50% of the total FE can be associated with the liquid ECR products (assuming negligible losses). We previously quantified several of these liquid products and established that the FE of C_2+_ liquids such as ethanol and propanol is related to that of ethylene and ethane in the gaseous stream. Especially, the FE of propanol was observed to significantly increase with increasing ethane content.^60^ Thus, a total FE of ∼80% can be realized for all the ECR products. If ethane, ethanol, and propanol were the only chemicals produced, then the H_2_ liberated, which accounts for about 20% of the total FE, would be sufficient to meet the H requirements of these products. Therefore, the 4.5 bar pressure may be considered optimal to effectively suppress HER, provided sufficient C_2+_ selectivity is observed. A further increase in pressure increases the CO yield. Based on the activity and selectivity of other catalysts, other pressure conditions might be necessary to achieve similar C_2+_ FEs.

Fig. 8(b) demonstrates the FEs of several catalysts including unstructured Cu(0), Cu(i), and Cu(ii) as well as femtosecond laser structured s-Cu(0), s-Cu(*x*), and s-Cu(ii) catalysts measured at 4.5 bar. As evident from Fig. 8(b), the unstructured Cu(0) mostly produces H_2_ along with some CO and methane. Laser structuring enhances the C_2+_ selectivity of s-Cu(0), leading to the production of ethylene instead of methane, which is completely suppressed after structuring. In contrast to Cu(0), unstructured Cu(i) produces significant amounts of both methane and ethylene as well as some traces of CO. Also, the HER is relatively suppressed compared to both Cu(0) and s-Cu(0). This emphasizes the role of oxidation state in influencing the selectivity of the Cu-based catalyst. Upon femtosecond laser structuring of Cu(0) in air, we get s-Cu(*x*) with the highest C_2+_ selectivity, also yielding ethane along with ethylene, whereas methane is totally suppressed. Furthermore, the HER is also significantly suppressed on s-Cu(*x*). In contrast to unstructured Cu(i), Cu(ii) can only produce CO besides H_2_. Changes in the oxidation state and atomic coordination are the most probable causes for this shift in selectivity. To analyze if structuring can improve the performance of Cu(ii) oxide, s-Cu(ii) was analyzed and was found to produce some ethylene along with H_2_ and CO. The ethylene production on s-Cu(ii) (decorated with a thick forest of CuO nanowires on top of the laser-induced nanostructures) (Fig. 4(c and d)), compared to unstructured Cu(ii) (with less dense nanowires and no laser structuring) (Fig. S4(b)†) underscores the significance of nanostructuring that exposes high-index facets and/or undercoordinated sites for prolonged adsorption of the intermediates. This finding aligns well with studies demonstrating that high-index facets on Cu surfaces facilitate the formation of multi-carbon products during ECR.^27^

Some researchers declare C_2+_ selectivity exclusively to Cu(i), often overlooking the role of Cu(ii) altogether.^17,18,20–23^ Compared to Cu(0) and Cu(ii), Cu(i) boasts intermediate adsorption strength for reaction intermediates while also facilitating delocalized d-band electrons, which likely explains this conclusion. However, the results reported here, provide convincing experimental evidence that low coordinated and well-dispersed mixed oxidation states involving Cu(0), Cu(i), and Cu(ii) are important for realizing C_2+_ products. The production of ethane with total suppression of methane on s-Cu(*x*) is an indication of the synergetic effects of low coordinated Cu(0), Cu(i), and Cu(ii) in promoting C_2+_ selectivity. We believe that the low coordinated Cu(i) and/or Cu(ii) provide the necessary adsorption strength to stabilize the intermediates, allowing them to remain adsorbed long enough for charge transfer to take place. However, the charge transfer is favorably facilitated by the relatively more electron rich d-band Cu(0) and Cu(i). Similarly, the relatively lower and moderate corresponding adsorption strengths of Cu(0) and Cu(i), which we believe are uniformly distributed along Cu(ii), promote hopping so that longer chains *via* C–C coupling could be realized. The Raman analysis discussed above (Fig. 5), also indicates the uniform distribution of Cu(i) within the Cu(ii) matrix. We observed strained broad peaks of Cu(ii) that were red shifted, with no single strong peak of Cu(i) in s-Cu(*x*). Lower coordination and such mixed oxide distributions are typical of femtosecond lasers, which deliver very high-power pulses with time durations on the order of femtoseconds.^60–67^

The above deductions regarding the optimized adsorption strength, intermediates' mobility, and charge transfer kinetics are in line with previous claims of efficient intermediate coupling observed on composite heterostructure catalysts. Such heterostructure catalysts may favorably adsorb specific intermediates due to the distinct chemical nature of the available active sites. The heterojunctions ensure close proximity of the active sites, thus reducing energy barriers for the coupling of intermediates to realize larger molecules. The heterostructures may also promote charge transfer kinetics because of variations in charge distribution along the heterojunction interface. Such spatial and electronic integration may suppress the competing HER by facilitating coupling at the interface.^68^

**Fig. 9:**
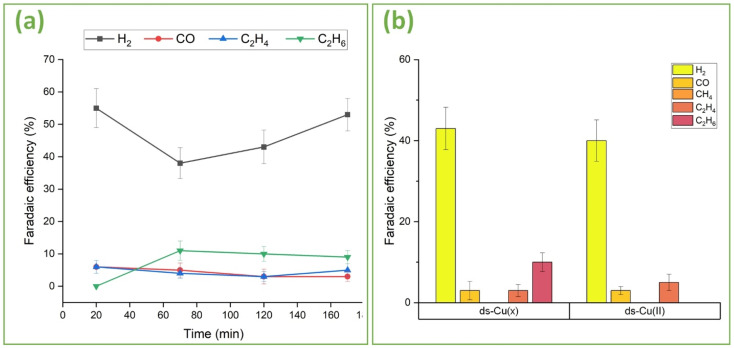
(a) Faradaic efficiency of ds-Cu(*x*) for gaseous products as a function of time. (b) Comparison of Faradaic efficiency of ds-Cu(*x*) and ds-Cu(ii) for gas products at 4 bar pressure.

To further unveil the effects of oxidation and surface structuring on C–C coupling, the dome-structured ds-Cu(*x*) and ds-Cu(ii) (Fig. 4) catalysts were analyzed (Fig. 9). Fig. 9(a) provides FE *vs.* time plots for ds-Cu(*x*). Similar to the above-discussed s-Cu(*x*), ds-Cu(*x*) can also effectively suppress methane and yield C_2+_ products including ethane, although with lower FEs compared to s-Cu(*x*). Similarly, the HER is also less suppressed compared to s-Cu(*x*). Furthermore, the FEs of most of the products including H_2_, are not constant but rather change with time. The dome structured Cu(*x*) was produced using a set of different laser parameters compared to s-Cu(*x*), resulting in different morphology and chemistry compared to s-Cu(*x*). It is less nanorough (Fig. 3(b)*vs.*Fig. 4(a)) than s-Cu(*x*), which may suggest higher atomic coordination. Similarly, it is apparently more oxidized (Fig. S8†*vs.*Fig. 2(k)) than s-Cu(*x*), hinting at a larger proportion of Cu(ii) (>81%) and a lower content of Cu(0) + Cu(i) (<19%) than on s-Cu(*x*). From the preceding discussion, we have discovered that mixed and well-dispersed oxides help boost the C_2+_ selectivity. With lower Cu(0) and Cu(i) content, the likelihood of intermediate adsorption (on Cu(i)) and efficient electron transfer (by both Cu(0) and Cu(i)) is reduced compared to s-Cu(*x*) and thus C_2+_ products are less likely on ds-Cu(*x*) than on s-Cu(*x*). For these reasons we observe relatively lower C_2+_ selectivity on ds-Cu(*x*). Since methane, which has the highest H : C ratio, was effectively suppressed, and other C_2+_ hydrocarbons such as ethane, which also have high H : C ratios, could not be produced efficiently, the HER consequently dominates. It is for these reasons that the FE of HER is quite high on ds-Cu(*x*). Furthermore, the change in selectivity with time is attributed to *in situ* changes in the oxidation state observed in this study, along with local environmental changes and surface reconstruction during ECR, as reported by others.^69–71^ The observed nonproportional changes in FE over time between the reported C_2+_ gas products and H_2_ are not necessarily correlated, as liquid products such as formate, methanol, ethanol, and propanol were not explored in this study.

Fig. 9(b) provides FE comparison data of ds-Cu(*x*) and ds-Cu(ii) after two hours of operation at −10 mA cm^−2^. Although both successfully suppress methane, ds-Cu(ii) is incapable of producing ethane. Their nano-morphologies (Fig. 4(a)*vs.*(b)) reveal that although ds-Cu(ii) has a very dense forest of CuO nanowires covering it, ds-Cu(*x*) is more selective towards longer C_2+_ products. It can be inferred that mixed oxides, even though less nanostructured, exhibit greater selectivity towards C_2+_ products than highly nanostructured Cu(ii).

## Conclusion

This study demonstrates the synergistic influence of reaction pressure, oxidation state, and catalyst morphology on C_2+_ selectivity of Cu-based electrocatalysts for electrochemical CO_2_ reduction (ECR). Using femtosecond laser structuring and thermal treatments, we synthesized Cu(0), Cu(i), Cu(ii), and mixed oxidation state Cu(*x*) catalysts exhibiting unique micro- and nano-morphologies. Thermally produced Cu(i) was confirmed as the only oxidation state among Cu(0), Cu(i), and Cu(ii), capable of yielding ethylene without structuring. However, nanostructuring enabled all the three oxidation states to yield C_2+_ products. Notably, nanostructured Cu(*x*) (*i.e.*, the mixed oxidation state catalyst) was found to produce ethylene as well as ethane with a combined Faradaic efficiency (FE) of 31% at 4.5 bar, while completely suppressing methane production. The enhanced C_2+_ selectivity of structured Cu(*x*) was attributed to the cooperative interaction of the well-dispersed and undercoordinated Cu(0), Cu(i), and Cu(ii) in promoting C–C coupling. Among the mixed states, Cu(i) and Cu(ii) provide the necessary adsorption strength to stabilize the reaction intermediates, allowing them to remain adsorbed long enough to facilitate coupling. Due to the more filled d-orbitals, charge transfer is more favored on Cu(0) and Cu(i) relative to Cu(ii) with less filled d-orbital. Similarly, the relatively lower adsorption strength of Cu(0) and Cu(i) helps in the hopping of reaction intermediates so that longer chains *via* C–C coupling could be realized. Lastly, elevating CO_2_ pressures up to 4.5 bar was also witnessed to improve C_2+_ yield, with combined gaseous C_2+_ FEs increasing by ∼200% relative to 1 bar, while suppressing H_2_ FE by ∼56%. The findings presented here enrich our understanding of the key factors influencing multicarbon product selectivity and provide important insights for the design of advanced Cu-based electrocatalysts for selective CO_2_ reduction.

## Data availability

For access to the datasets used and/or analyzed in this study, please contact the corresponding author.

## Author contributions

Asghar Ali: conceptualization, writing the original draft, methodology, investigation, formal analysis, data curation. Ali. S. Alnaser: conceptualization, review & editing, supervision, project administration, funding acquisition.

## Conflicts of interest

The authors have no conflict to declare.

## Supplementary Material

NA-OLF-D4NA01019A-s001
