# Supplementary material for: Unraveling the roles of pressure, oxidation state, and morphology in CO2 electroreduction to C2+ gaseous products over copper oxides
Source: Nanoscale Adv. 2025 Feb 25;7(8):2309–21. doi: 10.1039/d4na01019a (PMC11873738; doi:10.1039/d4na01019a)
Supplement: NA-007-D4NA01019A-s001 [file NA-007-D4NA01019A-s001.pdf]

This journal is © The Royal Society of Chemistry 2025

Supporting Information

Unraveling the Roles of Pressure, Oxidation State, and Morphology in CO_2 Electroreduction to C_{2+} Gaseous Products over Copper Oxides

Asghar Ali[1,2], Ali S. Alnaser[1,2,3*]

[1]Materials Science and Engineering Program, Materials Research Center, College of Arts and Sciences, American University of Sharjah, Sharjah, UAE

[2]Department of Physics, College of Arts and Sciences, American University of Sharjah, Sharjah, UAE

[3]Materials Research Center, American University of Sharjah, Sharjah, UAE

[*]Email: aalnaser@aus.edu

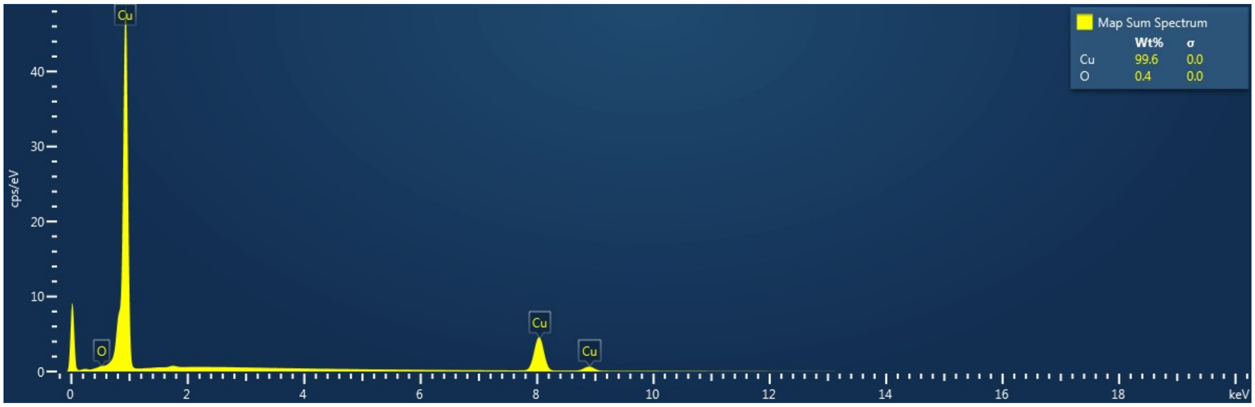

Figure S1. Point EDS pattern of unstructured Cu(0) as synthesized by polishing of metallic Cu (no thermal treatment)

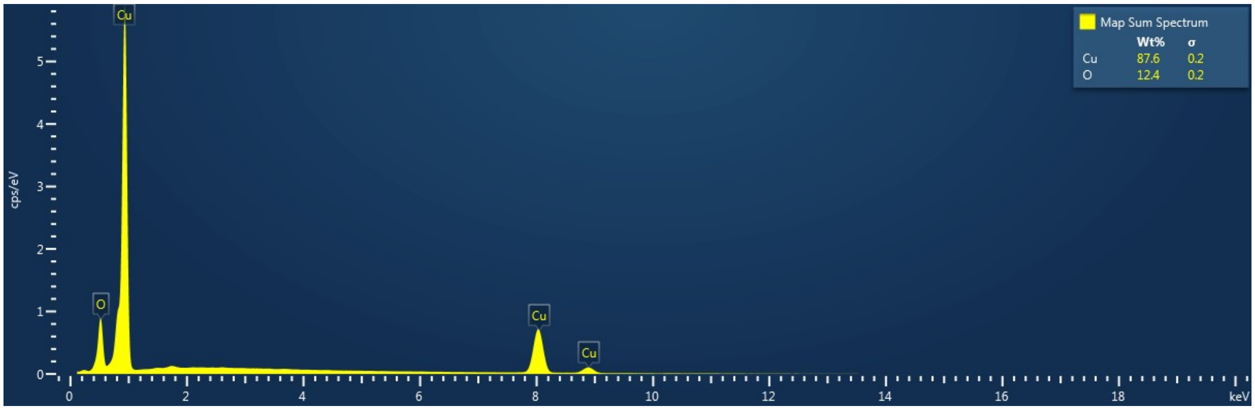

Figure S2. Point EDS of unstructured Cu(I) synthesized via thermal treatment of polished Cu at 220 ℃ in air

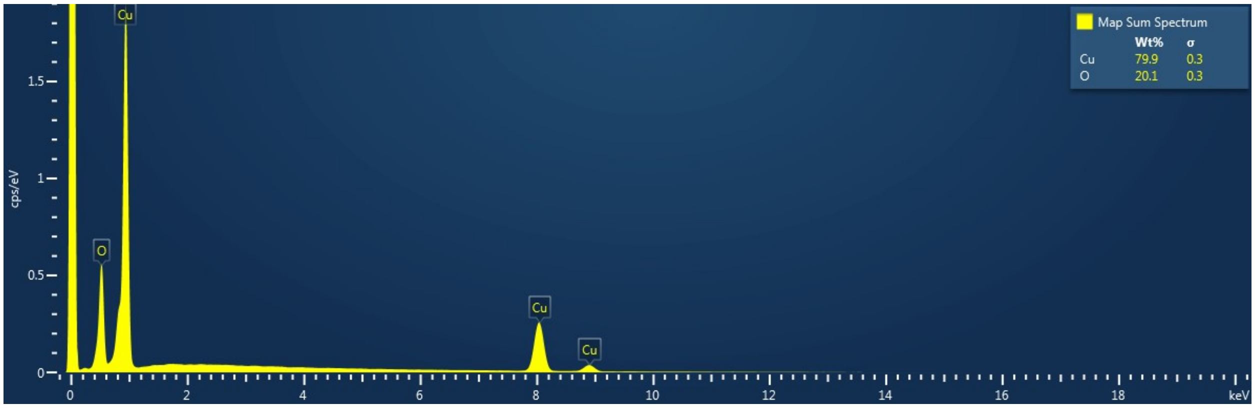

Figure S3. Point EDS of unstructured Cu(II) synthesized via thermal treatment of polished Cu at 350 ℃ in air

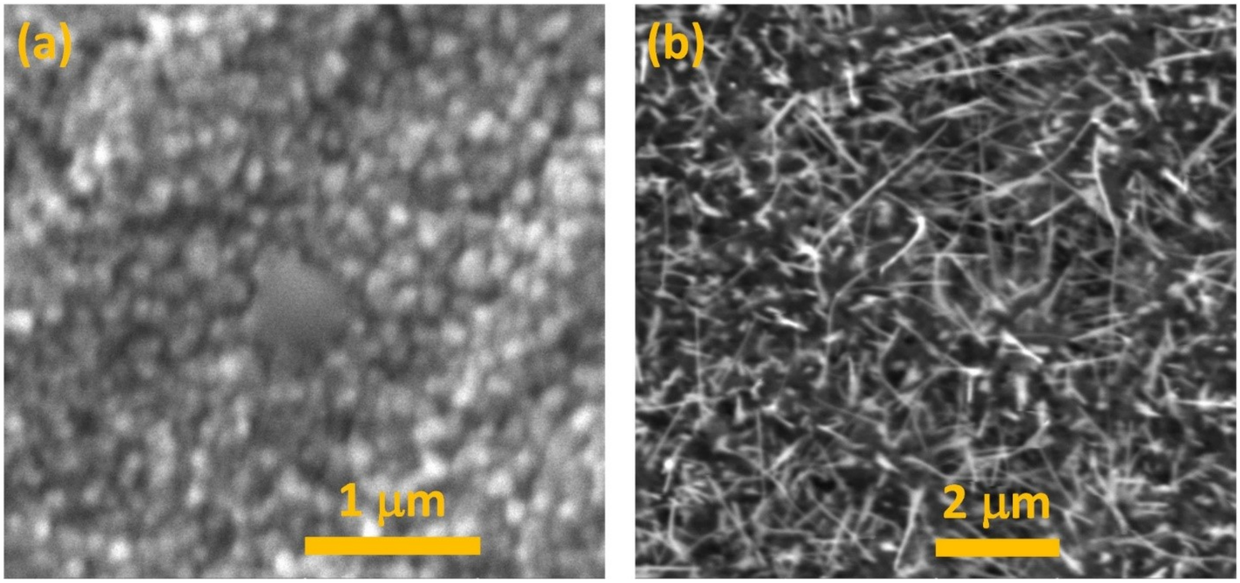

Figure S4. SEM micrographs of unstructured Cu after thermal treatment at 220 ℃ and 350 ℃ in air

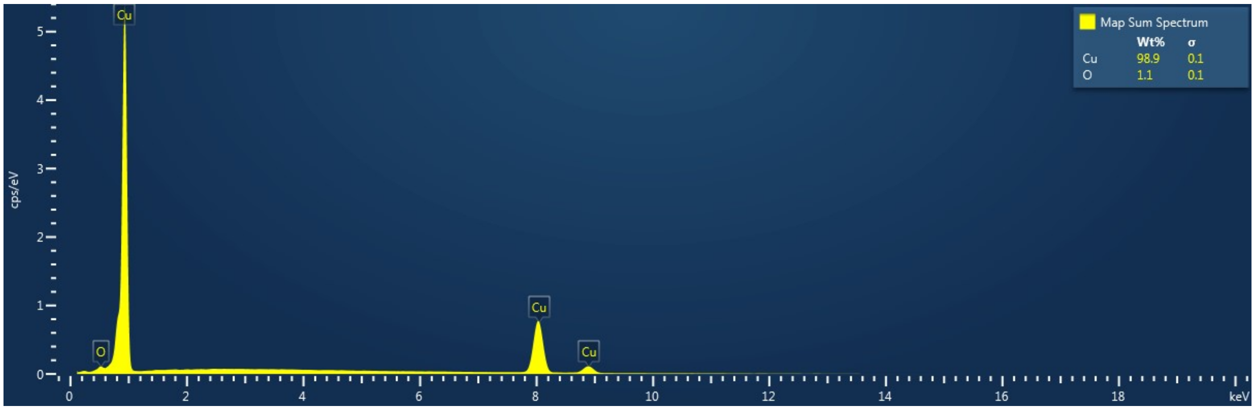

Figure S5. EDX spectrum of as synthesized structured Cu(0). Structuring was done with a single femtosecond laser scan at 5 W, 10 mm/s scan speed, and 20 μm line spacing in parallel configuration in Ar environment.

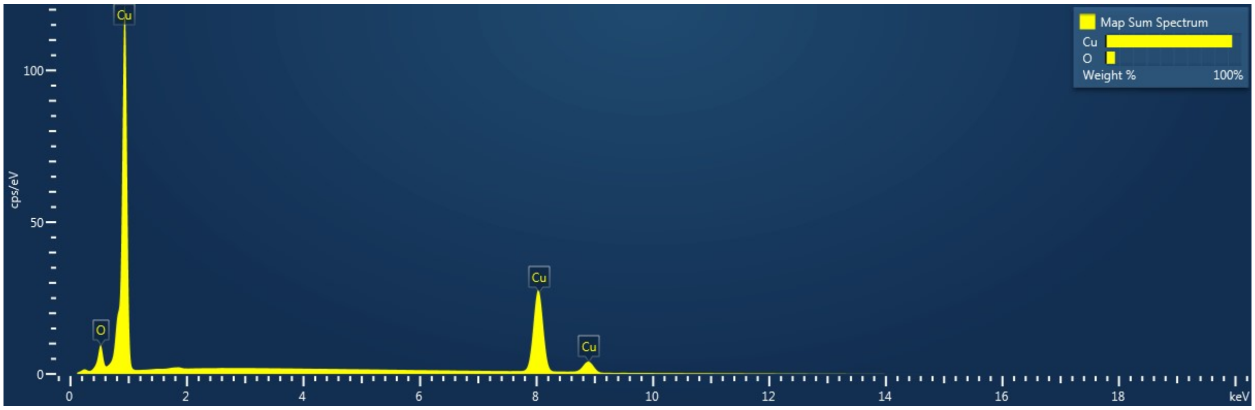

Figure S6. EDX spectrum of laser structured Cu(x) (substoichiometric) without post thermal treatments. Structuring was done with a single femtosecond laser scan at 5 W, 10 mm/s scan speed, and 20 μm line spacing in parallel configuration in air.

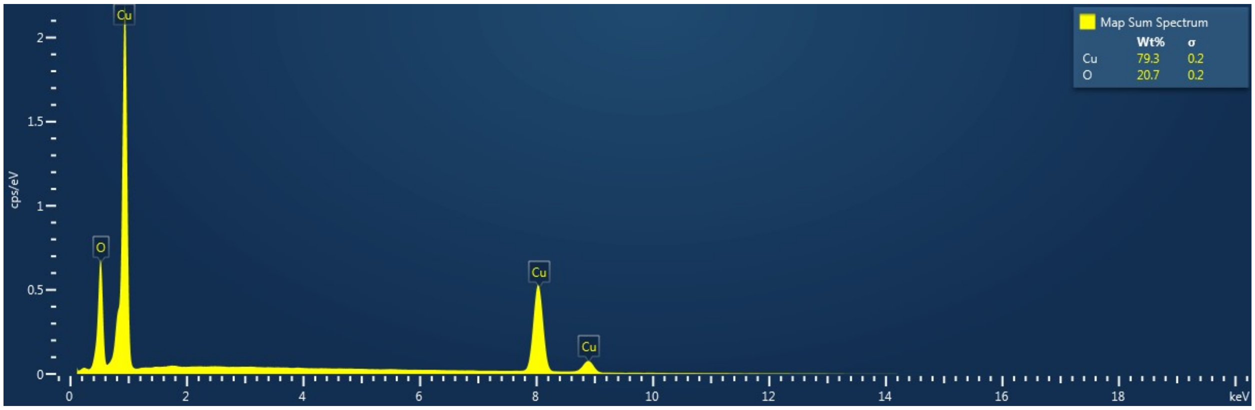

Figure S7. EDX spectrum of structured Cu(II) thermally treated at 350 ℃. Structuring was done with a single femtosecond laser scan at 5 W, 10 mm/s scan speed, and 20 μm line spacing in a parallel configuration in air.

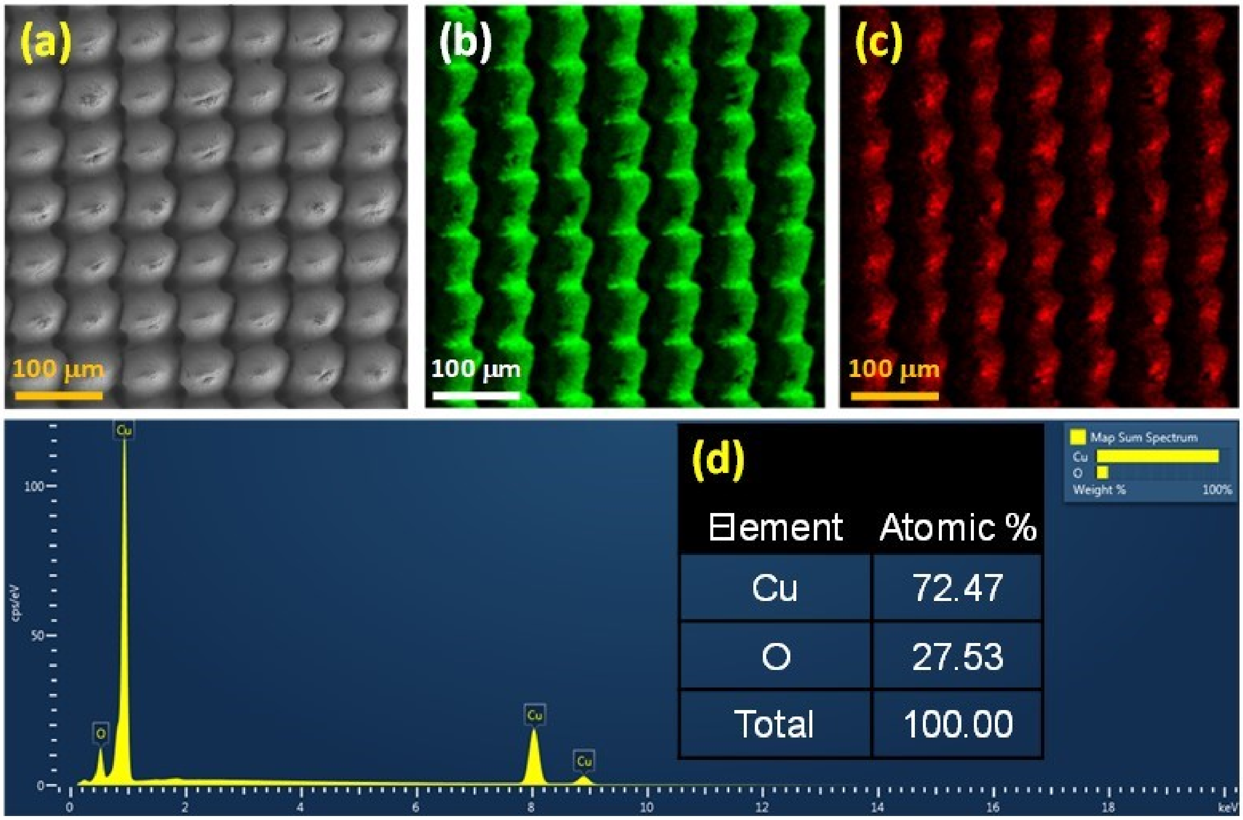

Figure S8. (a) SEM micrograph, (b, c) EDX maps showing the distribution of (b) Cu and (c) O along the surface in (a), and (d) the corresponding EDS pattern with elemental composition of ds-Cu(x). ds-Cu(x) was fabricated by femtosecond laser structuring of Cu(0) with a 70 μm line spacing in a cross-hatch configuration without any subsequent thermal treatment.

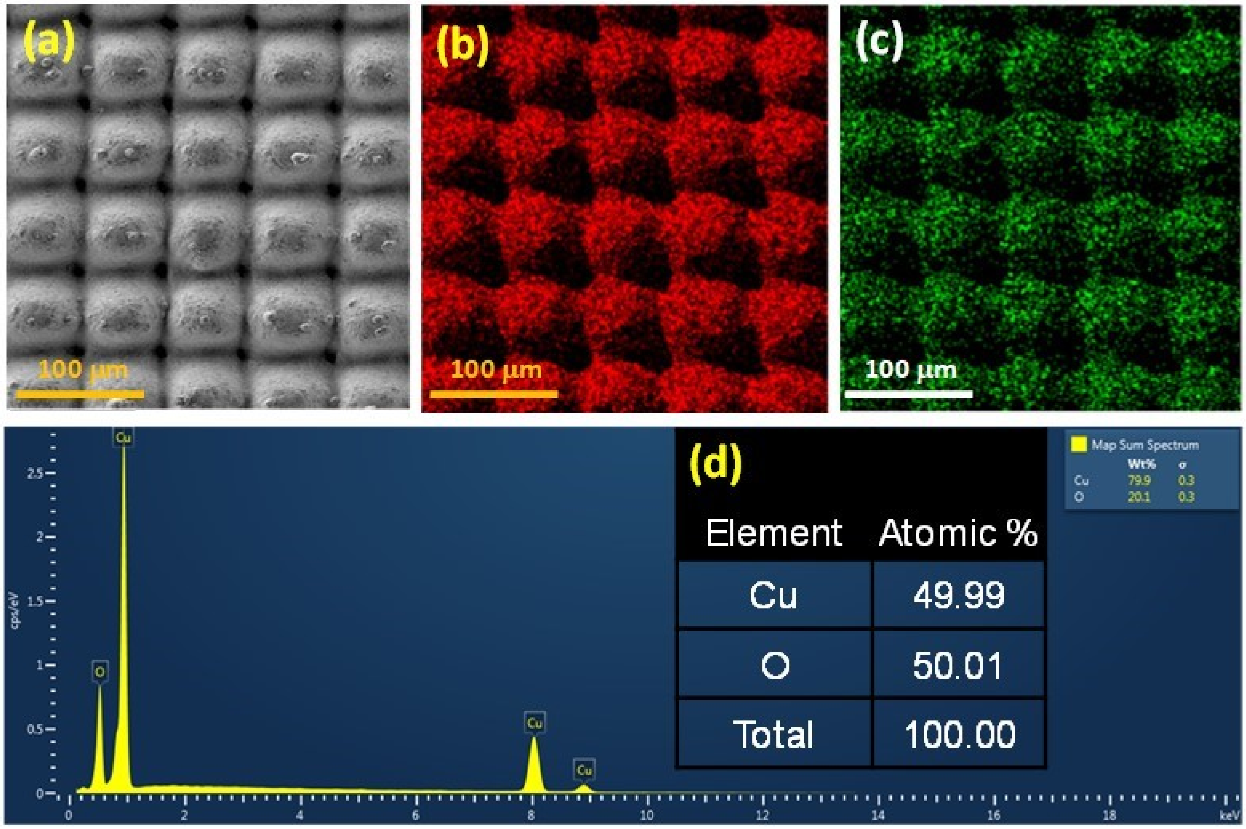

Figure S9. (a) SEM micrograph, (b, c) EDX maps showing the distribution of (b) O and (c) Cu along the surface in (a), and (d) the corresponding EDS pattern with elemental composition of ds-Cu(II). ds-Cu(II) was fabricated by femtosecond laser structuring of Cu(0) with a 70 µm line spacing in a cross-hatch configuration, followed by thermal annealing in air at 350 ℃.
